# Hysteropexy and Anterior Vaginal Native Tissue Repair in Women with Anterior and Central Compartment Prolapse: A Long Term Follow-Up

**DOI:** 10.3390/jcm12072548

**Published:** 2023-03-28

**Authors:** Maurizio Serati, Stefano Salvatore, Marco Torella, Chiara Scancarello, Andrea De Rosa, Alessandro Ferdinando Ruffolo, Giorgio Caccia, Fabio Ghezzi, Andrea Papadia, Yoav Baruch, Andrea Braga

**Affiliations:** 1 Department of Obstetrics and Gynecology, Del Ponte Hospital, University of Insubria, 21100 Varese, Italy; 2 Department of Obstetrics and Gynecology, IRCSS San Raffaele Scientific Institute, 21132 Milan, Italy; 3 Department of Gyanecology, Obstetric and Reproductive Science, Second University of Naples, 80138 Naples, Italy; 4 Department of Obstetrics and Gynecology, EOC—Beata Vergine Hospital, 6850 Mendrisio, Switzerland; 5 Department of Obstetrics and Gynecology, EOC—Civico Hospital, 6900 Lugano, Switzerland; 6 Faculty of Biomedical Sciences, Università della Svizzera Italiana, 6900 Lugano, Switzerland; 7 Department of Obstetrics and Gynecology, Tel Aviv Medical Center, Tel Aviv University, Tel Aviv 6997801, Israel

**Keywords:** hysteropexy, cystocele, pelvic organ prolapse, apical prolapse, transverse cystocele repair

## Abstract

Although it is known that hysterectomy (HY) alone cannot resolve apical prolapse, vaginal hysterectomy (VH) remains the most common surgical procedure for this issue. In recent years, various procedures for uterine conservation have been proposed to avoid the surgical risks of HY. Furthermore, most women with symptomatic pelvic organ prolapse (POP) prefer uterine conservation in the absence of considerable benefit in uterine removal. In 2017, we proposed a new technique for hysteropexy and anterior vaginal native tissue repair (NTR) in women with cystocele and apical prolapse. The objective of this study is to assess the efficacy and safety of this new procedure after at least 5 years of follow-up. We included only patients with stage II or greater prolapse of the anterior vaginal wall and a concomitant stage II uterine prolapse in accordance with Pelvic Organ Prolapse Quantification (POP-Q) system. A Patient Global Impression of Improvement (PGI-I) score ≤ 2 in addition with the absence of POP symptoms was defined as subjective success. A descensus with a maximum point of less than −1 in any compartment was considered objective cure. A total of 102 patients who fulfilled the inclusion criteria were enrolled. At 60 months follow-up, 90 out of 102 patients (88%) were subjectively cured, whereas 88 out of the 102 (86%) patients were objectively cured. Subjective and objective cure rates persisted during the entire study period. Uni- and multivariate analysis of possible predictive factors associated with recurrence of prolapse showed that only a preoperative point C > 0 cm and BMI ≥ 25 kg/m^2^ were risk factors for failure. In conclusion, our study showed that hysteropexy with anterior vaginal native tissue repair may be an effective and safe option for the treatment of anterior vaginal prolapse and concomitant stage II uterine prolapse by at least 5 years of follow-up.

## 1. Introduction

The uterosacral cardinal ligament structure is the main support for the uterus. Weakness or defects in this structure result in utero-vaginal prolapse.

It is well demonstrated that HY alone is not able to resolve apical prolapse [1], however, VH remains the most common surgical procedure for this condition. 

In fact, uterine prolapse is the most frequent indication for HY in women over 50 years of age [2]. It accounts for about 20% of hysterectomies performed each year in the United States (US) [3]. 

In recent years, various procedures for uterine conservation have been proposed to avoid surgical risks of HY, to maintain fertility, to preserve sexual satisfaction and a sense of identity [4]. Furthermore, most women with symptomatic POP prefer uterine conservation in the absence of considerable benefit in uterine removal [5]. Several studies have shown no significant differences in terms of recurrence rates, quality of life (QoL) and urogenital symptoms between HY and procedures preserving the uterus at 12 months of follow-up [6,7]. 

However, due to a lack of training in uterine-sparing surgical procedures, hysteropexy procedures are poorly considered by pelvic surgeon. They mistakenly believe that concomitant HY leads to better surgical results and that future HY may be more technically demanding [4]. 

Serati et al. in 2017 [8] proposed a new technique for hysteropexy and anterior vaginal native tissue repair in women with cystoceles and apical prolapses. The authors demonstrated that their procedure had a low morbidity and blood loss, short operating time and minimal postoperative and intraoperative complications. They found an objective and overall cure rate of 95.1% at 30 months of follow-up.

The primary outcome of this study is to assess the efficacy and safety of this new procedure of vaginal cystocele repair and hysteropexy by at least 5 years of follow-up. The secondary outcome is to evaluate potential risk factors related to POP recurrence.

## 2. Materials and Methods

This is an update of the previous study [8] performed in our urogynaecological division at the University of Insubria in Varese, Italy. Between January 2013 and June 2016, we prospectively enrolled all consecutive patients with any prolapse symptoms and/or who experienced a sense of “vaginal bulge” or a “lump of fullness in the vagina”. Preoperatively, written informed consent was signed by each patient. We included only patients with stage II or greater prolapse of the anterior vaginal wall and a concomitant stage II uterine prolapse in accordance with the POP-Q system [9]. The severity of symptoms was recorded using a visual analogue scale (VAS) (from 0 to 10 points, with 0 meaning no impact and 10 meaning the worst imaginable impact). Preoperative assessment included clinical history, physical evaluation, a 3-day bladder diary, urine analysis, pelvic ultrasound examination and urodynamic study (UDS). Physical evaluation was carried out with the patient in the gynaecological position and POP was examined during a maximal Valsalva manoeuvre as described by the POP-Q system [9]. We excluded patients with an elongation of the cervix that simulates a prolapse. As previously described [10], UDS was performed by an expert urogynaecologist, using a standardized protocol according to the Good Urodynamic Practice Guidelines of the International Continence Society (ICS) [11]. As reported in a previous study [8], we excluded: abnormal uterine bleeding, endometrial anomalies on ultrasound and/or biopsy, a posterior vaginal wall prolapse greater than stage I, uterine prolapse in case of C point > +1, previously performed anti-prolapse surgical procedures and/or concomitant abdominal surgical approach for pelvic disease. All surgical procedures, which consisted of a modified version of the technique described by Huffaker et al. [12], were performed by the same surgeon with extensive experience in urogynaecological surgery. A urinary catheter and a gauze in the vagina were placed at the end of surgery for one night. After removal of the catheter, an ultrasonographic examination of the postvoid residual was performed. Furthermore, a urine culture was carried out. Postoperative examinations were mandatory at 3 and 12 months after surgery and then were scheduled for every year. The follow-up examinations were performed by two urogynaecological physicians who had not performed the surgical procedures. Every follow-up evaluation included: physical examination, urinalysis and the POP quantification. To investigate the subjective outcome, all women completed the PGI-I scale (a 7-point scale, with a range of responses from 1, ‘‘very much improved’’, through 7, ‘‘very much worse’’) [13]. A PGI-I score ≤ 2 (“very much improved” or “much improved”) in addition to the absence of POP symptoms was considered a subjective success. We defined the objective cure as a prolapse with a maximum point of less than −1 in any compartment. Several randomized trials (RCTs) considered the absence of symptoms of prolapse, a PGI-I score ≤ 2, prolapse stage < II, and no need for other surgery as overall success [14]. All women completed the Prolapse Quality of Life (P-QOL) questionnaire [15,16] pre- and postoperatively and subsequently at every follow-up visit. In the P-QOL questionnaire, the responses range from 0 “none/not at all”, through “slightly/a little” and “moderately”, to 100 “a lot”. A higher total score intends a greater impairment of QoL, while a lower total score indicates a better QoL. Ethical approval was obtained from our Institutional Review Board before the start of the study.

### Surgical Technique

After preoperative examination and instillation of one vial of methylene blue into the bladder, a midline longitudinal colpotomy was performed from the ureterovesical junction to the anterior lip of the cervix. The vaginal epithelium was dissected away from the underlying pubocervical connective tissue and bladder. The anterior wall of the uterine cervix was also dissected from the base of the bladder to expose the entire anterior isthmocervical portion. This portion was grasped bilaterally with two pieces of Vicryl 0.0 suture. The midline plication of the pubocervical connective tissue was then performed. To close the transverse defect and to suspend the isthmus of the uterus at the endopelvic tissue, the two previously inserted suspensory sutures in the cervical stroma to the side were attached to the repaired pubocervical fascia. If necessary, the vaginal epithelium was trimmed. Anterior colporrhaphy was completed with a series of simple side-to-side interrupted Vicryl 2.0 sutures.

## 3. Statistical Analysis

We used SPSS for Windows, version 17 (SPSS, Chicago, IL, USA) and GraphPad version 6 (GraphPad Software, San Diego, CA, USA) for the statistical analysis. The chi-squared and Fisher’s exact tests were applied to analyse proportions. We used Student’s *t*-test and the Mann–Whitney U test to compare continuous parametric and nonparametric variables, as appropriate. The chi-squared and the chi-squared test for trend were used to analyse and compare surgical results. The chi-squared test is the most appropriate test to evaluate the success of surgical interventions to decrease over time by comparing the cure rates at the different follow-up visits (1 year, 2 years, 3 years and 5 years). The null hypothesis was to find no significant correlation between the cure rate of this surgical procedure and time. The Cox proportional hazards model was performed to assess factors probably impacting on the risk of recurrence during the follow-up period. 

## 4. Results

One hundred and four patients who met the inclusion criteria were enrolled. We excluded two women because they did not want surgical treatment. During the study period, all patients underwent modified transverse cystocele repair with hysteropexy as described by Serati et al. [8]. Characteristics of patients and their preoperative UDS data at baseline, are shown in Table 1.

A total of 49% of patients had stage II, 28% had stage III and 27% had stage IV of anterior compartment prolapse at the baseline, while all patients had stage II of apical prolapse. Surgical data are reported in Table 2. No intraoperative complications were noted. Only five (4.9%) and three (2.9%) patients reported early and late postoperative complications, respectively (Table 3).

No patients reported considerable postoperative pain and long-term complications at the last available follow-up. The mean follow-up was 64 (60–71) months (mo) and no patient was lost to follow-up. At 5 years follow-up, 90 out of the 102 patients (88%) were subjectively cured (*p* for trend 0.005), whereas 88 out of the 102 (86%) patients were objectively cured (*p* for trend 0.02). Subjective, objective and composite cure rates did not change during the entire follow-up (Table 4).

Subjective anatomical prolapse recurrence was reported in 12 (12%) patients. Among these, only three women required another surgical procedure including an anterior colporrhaphy plus VH and McCall Culdoplasty. All patients had anterior vaginal prolapse with Ba point at 1 cm, while C point was >1 cm in two patients and >2 cm in one patient. Regarding objective outcomes, another two women (1.9%) reported asymptomatic recurrence in the cystocele (point Ba 0 cm in both). During the follow-up period, we had four (4%) pregnancies. All patients underwent Caesarean section (CS) without POP recurrence. The Ba and C point accordance with POP-Q measurements at baseline, at 12-mo and at last available follow-up examination are reported in Table 5. No statistical differences were found in other compartments in terms of POP-Q points. Furthermore, we found a statistically significant improvement for all domains of the P-QOL questionnaire.

The univariate analysis of potential risk factors related to POP recurrence found that only a preoperative point C > 0 cm and BMI > 25 kg/m^2^ were risk factors for failure (Table 6). Multivariate analysis confirmed the following predictive risk factors for recurrence of POP: preoperative point C > 0 (OR: 2.2 (95% CI: 1.4–3.8); *p* 0.01) and BMI ≥ 25 kg/m^2^ (OR 1.8 (95% CI: 1.1–2.8); *p* 0.04

## 5. Discussion

Our study demonstrates the subjective and objective results of the hysteropexy with anterior vaginal native tissue repair undergone by patients with a predominant cystocele and a concomitant apical prolapse of stage II. We found that this new surgical technique is a highly effective and safe procedure, with a long-lasting efficacy.

As reported by De Lancey [1], as long as the level I suspensory support is intact (cardinal and uterosacral ligament complex), apical prolapse does not occur. In women for whom HY was performed without a guarantee of the reattachment of the suspensory apparatus, the risk of subsequent vaginal prolapse may be increased [1].

For this reason, it is important to consider whether HY is necessary to adequately repair uterovaginal prolapse, taking into account that in the US more than 100,000 hysterectomies are performed every year for POP [17]. After removal of the uterus, about 12% of patients need another surgical repair for prolapse, and in two-thirds of these cases a multicompartment repair is required [18].

Uterine conservation during reconstructive pelvic surgery is an option that is increasingly considered by women who have POP [4]. In fact, previous studies have shown that many women with POP prefer to maintain their uterus in the absence of a considerable benefit to HY [19]. Uterine-sparing procedures have the advantages of reducing blood loss, operative time and mesh exposure compared with similar surgical routes with concomitant HY [20].

In fact, despite the highly satisfying anatomical result and the encouraging recurrence rate, the use of mesh is associated with a high number of mesh-related complications, such as erosions, infections, shrinkage with vaginal shortening resulting in chronic pain and dyspareunia. As stated by leading international scientific societies, the use of these synthetic devices requires caution and should be used only for recurrent POP or high-risk patients for whom the benefit of mesh may justify the risk for this procedures [18]. Several governments, including Australia, New Zealand, the United Kingdom and US, have banned the use of transvaginal mesh (TVM) for POP repair [18].

As demonstrated by Maher et al. in a Cochrane review on surgery for apical vaginal prolapse [21], the evidence does not sustain the use of TVM compared to NTR for apical prolapse. The authors found no significant differences in terms of subjective and objective outcomes and reoperation rate for recurrence at a minimum of 2 and a maximum of 4 years follow-up.

Furthermore, independently of the route used to insert the mesh (vaginal, abdominal, laparoscopic or robotic), it is more frequently associated with serious complications [21,22]

In conservative pelvic surgery, after the criticisms related to the mesh complications, NTR procedures are progressively regaining an important role, with many advantages as well as a low incidence of complications [23].

As reported in a recent systematic review and meta-analysis by Meriwether et al. [20], transvaginal uterosacral or sacrospinous hysteropexy (SSH) or the Manchester procedure (MP) in comparison with VH with NTR both showed better outcomes in terms of surgical time and bleeding without a worsening of results with uterine conservation. However, data on the outcomes of POP over 3 years after surgery are lacking.

An observational follow-up of SAVE U (SSH vs. VH in treatment of uterine prolapse ≥2) randomised controlled trial (RCT) [6,24] showed that at 5 years follow-up, uterine conservation is more effective than VH with uterosacral ligament suspension (USLS), 87% vs. 76% in composite outcomes (defined as no POP > 0, no symptomatic POP, and no reoperation or pessary use for recurrent POP), respectively. Subjective failure of the central compartment or repeat surgery occurred in one patient (1%) after SSH compared with eight patients (7.8%) after VH with USLS (−6.7%, 95% CI −12.8% to−0.7%). 

Recently, MP is also increasing in popularity. Ünlübilgin et al. [25], in the only RCT published in the literature that compared the MP to VH for uterine prolapse, found no difference in terms of prolapse recurrence (defined as POP-Q point C ≥ −1), QoL and serious complications, at 5 years follow-up. However, MP had a shorter surgical time (*p* = 0.003) and length of stay (*p* = 0.042) than VH.

Furthermore, only one patient in the MP group underwent a second POP procedure during the study period compared with three patients in the VH group.

All other studies available in the literature, which have compared MP with VH with or without apical NTR, have a retrospective design and follow-up between 1 and 6 years. Although these studies are poor in terms of the quality of evidence, they suggest that MP may be advantageous over VH in a population that would benefit from limited surgical time and lower blood loss, with no change in POP recurrence rate [26].

The same advantages were reported by several authors when comparing uterosacral ligament hysteropexy (USLH) with HY [23]. However, there are no RCTs comparing outcomes of USLH and VH with uterosacral ligament suspension (USLS).

Most studies retrospectively compared USLH (laparoscopic or vaginal) to VH. Both approaches showed a similar efficacy and safety as VH, with a shorter surgical time and less blood loss [27,28]. Milani et al. [27] in a retrospective study compared 52 patients who underwent USLH to 52 matched control patients who underwent HY plus USLS. At 35 months of median follow-up, the authors showed that overall anatomic and subjective outcomes were similar between groups, as well as the rate of complications. USLH was associated with a shorter surgical time and less blood loss compared with VH. However, USLH was found to be associated with a higher apical recurrence rate (21.2% vs. 1.9%, *p* = 0.002), and subsequently a higher reoperation rate (13.5% vs. 1.9%, *p* = 0.04). As found in our study, the most important risk factor for recurrence was cervical elongation [27]. For this reason the preoperative selection of patient candidates for a hysteropexy procedure is important to guarantee long-term symptom-free survival recurrence.

In addition, the Sixth International Consultation on Incontinence on treatment of POP [29] considered hysteropexy a reasonable option for the primary treatment of uterovaginal prolapse, however, the long-term data are poor and the need for subsequent HY unknown.

Another advantage for uterus conservation is uterus-sparing procedures. In fact, the data regarding pregnancy after uterus-sparing procedures are promising. In a small retrospective case series of women who underwent MP, 28.6% subsequently became pregnant and had a successful vaginal delivery [30]. Singh et al. [31], in a retrospective observational study on MP, reported a 7.3% of pregnancy rate (two women underwent CS at 36 weeks, one woman had an emergency CS at 28 weeks for preterm premature rupture of the membranes, and the last woman voluntarily terminated her pregnancy at 10 weeks).

In addition, SSH seems to be an adequate surgical procedure for symptomatic prolapse in patients who wish to conserve their fertility. Cavkaytar et al. reported 8 pregnancies in a series of 54 patients who underwent transvaginal SSH due to grade 2 uterine prolapse. All women delivered by elective CS by at least 37 weeks with only one preterm delivery (due to a twin pregnancy) [32]. As reported by Milani et al. [27] in their series on USLH, three out of seven patients (42.9%) became pregnant and delivered at term by elective CS. One patient terminated her pregnancy voluntarily.

Another aspect to take into account when considering uterus-sparing procedures is the impact of these on sexual function. There is a lack of studies in the literature that evaluate this aspect after hysteropexy. The only RCT available in the literature compared results of patients randomized to SSH or VH with sacrospinous ligament fixation [33]. Four domains were evaluated: sexual interest, sexual satisfaction, frequency of sexual intercourse and orgasm. The authors found no difference in terms of domain scores from preoperative to postoperative time points. Frequency of orgasm was the only domain that changed significantly after surgery in the two groups due to fear of wound disruption or disease recurrence. These reasons lead to patients refraining from vigorous or exciting intercourse.

Detollenaiere et al. published a secondary analysis of their RCT [6,34] focused on sexual function. They found no difference in overall sexual functioning evaluated by the Prolapse Incontinence Sexual questionnaire 12 (PISQ-12) between women who underwent SSLH vs. VH plus USLS after 24 months of follow-up. The authors further stated that a 60-mo follow-up of these subjects is planned to better clarify the long-term outcomes of SSH vs. VH.

Despite the benefits related to uterine conservation, pelvic surgeons hardly consider it as a viable option, because of the belief that it is more technically challenging [4].

Huffaker et al. in 2008 proposed a simplified procedure for transverse cystocele repair with uterine NTR in selected patients with stage 0 or I apical prolapse [12]. Although, the authors reported excellent anatomic success rates with no intraoperative complications, the relative short follow-up (median 6.1 weeks), represents a major limitation for its complete evaluation.

In 2017, we proposed a new technique for hysteropexy and anterior vaginal NTR that includes the closure of the transverse defect with cervical stroma and therefore uterine suspension in women with cystocele of stage II or higher and concomitant stage II uterine prolapse [8]. Our first experience with the use of this new technique showed a very low subjective failure rate (2% of patients). The objective cure rate and the overall cure rate were 95.1%. No significant deterioration in objective cure rate was observed over 30 months of follow-up.

Furthermore, the technique had minimal morbidity and low postoperative complications, a limited operating time, and seemed to be painless. In this study, we evaluated this procedure over a long-term period.

Our series seems to show that our technique is safe and highly effective for the treatment of cystocele (stage ≥ II) and concomitant stage II uterine prolapse, even in the long-term follow-up. In fact, we found 5-year subjective and objective cure rates of 88% and 86%, respectively, without any worsening in the cure rate over time. Only three women required a second surgical procedure including VH which was not complicated by the previous uterine fixation. It is important to highlight the very low rate of early and late postoperative complications and the absence of long-term consequences in terms of quality of life.

As previously demonstrated [8], and as evidenced by several studies that evaluated different hysteropexy procedures [7,35], a point C > 0 cm is the most important risk factor for recurrence. We also found that BMI > 25 kg/m^2^ is an independent risk factor for the failure of this procedure and symptomatic recurrence of POP. For these reasons, preoperative counselling is a crucial requirement to avoid false expectations and to inform patients about attitudes to be maintained in the pre- and postoperative period, especially regarding dietary intake. Our new procedure seems to be effective in cases of a predominant cystocele with low risk of anterior compartment. This could be related to the reattachment of the pubocervical fascia of the bladder base to Level I suspensory support by isthmocervical stroma of the uterus, ensuring the closure of the transverse defect.

Another advantage of our technique is the ability to preserve fertility. A total of 4 out of 102 patients became pregnant. All women underwent elective CS without any neonatal complications. Furthermore, it is important to highlight that none of these women showed any recurrence of prolapse during the follow-up period.

The strengths of this study are (1) the long-term evaluation; (2) the evaluation of subjective and objective outcomes obtained using validated tools and (3) the fact that no women were lost to follow-up. The limitation is that we did not evaluate sexual function in our population. Further research is needed to compare this intervention to other procedures.

## 6. Conclusions

The results of this study showed that hysteropexy with anterior vaginal native tissue repair may be an effective and safe option for the treatment of anterior vaginal prolapse and concomitant stage II uterine prolapse over at least 60 months. The procedure is painless with a very low rate of complications and recurrences.

Furthermore, this new technique is feasible in women who wish to preserve fertility for future childbearing.

## Figures and Tables

**Table 1:** Preoperative patients characteristics.

Preoperative Characteristic	*n* (%)
Age (years)	61.5 (42–69) *
Postmenopausal status	92 (90)
Hormone Replacement Therapy (HRT)	12 (12)
Birth weight > 4000 g	19 (19)
Vacuum delivery	6 (6)
Body mass index (kg/m^2^)	25.3 (23–28) *
Parity (mean)	2 ± 1 **
Previous POP surgery	9 (9)
Smoking habit	9 (9)
OAB symptoms	22 (21)
Urodynamic Stress Urinary Incontinence	26 (25)
Detrusor Overactivity	18 (18)

* IQR: interquartile range; ** SD: standard deviation.

**Table 2:** Surgical data.

Surgical Data	*n* (%)
Surgery time (min)	51 ± 14.7 *
Estimated blood loss (mL)	50 ± 18.6 *
Length of stay (days)	1 ± 0.2 *
Early postoperative complications	5 (5)
Late postoperative complications	3 (3)
Objective anterior recurrence	10 (10%)
Objective anterior and apical recurrence	4 (4%)
Subjective recurrence	12 (12%)
Pain on day 1 (VAS scale)	0 ± 1 *

* SD: standard deviation.

**Table 3:** Clavien–Dindo classification of early and late postoperative complications.

Early Postoperative Complication	*n =* 5	Action
CLAVIEN 1		
UTI	2 (25)	Antimicrobial prophylaxis or therapy
Voiding dysfunction	2 (25)	Observation and indwelling catheterisation
Fever (>38 °C)	1 (12.5)	Antipyretic therapy
		
Late postoperative complications	*n* = 3	
CLAVIEN 2		
Recurrent UTIs	1 (12.5)	Antimicrobial prophylaxis and therapy
De novo OAB	2 (25)	Antimuscarinics/β-agonists
		
UTI: urinary tract infection		

**Table 4:** Cure rates at the 12-months 36-months and 60-months follow-up visit.

Cure Rate	12 Months	36 Months	60 Months	*p* Value Chi-Squared Test	*p* Value Chi-Squared Test for Trend
**Objective**	98/102 (96%)	92/102 (90%)	88/102 (86%)	0.05	0.02
**Subjective**	100/102 (98%)	96/102 (94%)	90/102 (88%)	0.02	0.005
**Composite**	98/102 (96%)	92/102 (90%)	88/102 (86%)	0.05	0.02

**Table 5:** Anatomical and clinical results at 12 months and 60 months follow-up.

	Baseline	12 Months	60 Months	*p* Value Baseline vs. 12 Months	*p* Value Baseline vs. 60 Months
Ba	+0.5 ± 1.022	−2 ± 0.7	−2 ± 0.9	<0.0001	<0.0001
C	0 ± 0.53	−5 ± 1.2	−4 ± 1.5	<0.0001	<0.0001
VAS score symptoms	9.5 ± 1.7	0.2 ± 0.2	1.8 ± 1	<0.0001	<0.0001
PGI-I score ≤ 2		100/102	90/102		

**Table 6:** Univariate analysis of potential risk factors related to POP recurrence.

Factors	Subjective Recurrence HR (95% CI)	
Elderly (age ≥ 65 years)	0.61 (0.43–1.34)	0.3
Obesity (BMI ≥ 25 kg/m^2^)	1.99 (1.03–3.29)	0.04
Postmenopausal status	0.71 (0.44–1.70)	0.2
HRT	1.43 (0.62–2.31)	0.4
Point C > 0 cm	1.88 (1.20–3.23)	0.02
Previous POP surgery	0.31 (0.16–2.43)	0.5

Univariate Cox proportional hazard model.

## Data Availability

Data available on request due to privacy restrictions.
